# Evaluation of Sub-acute toxicity and safety profile of Charmagaz seed oil in rats

**DOI:** 10.1371/journal.pone.0327697

**Published:** 2025-07-11

**Authors:** Anum Adil, Saira Saeed Khan, Sadaf Naeem, Ali Alqahtani, Taha Alqahtani, Ali Asgher Shuja, Anosh Tahir

**Affiliations:** 1 Department of Pharmacology, Faculty of Pharmacy, University of Karachi, Karachi, Pakistan; 2 Division of Endocrinology, Diabetes and Metabolism, Department of Medicine, College of Medicine and Life Sciences, University of Toledo, Toledo, Ohio, United States of America; 3 Institute of Pharmaceutical Sciences, Jinnah Sindh Medical University, Karachi, Pakistan; 4 Department of Pharmacology, College of Pharmacy, King Khalid University, Guraiger, Abha, Saudi Arabia; 5 H.E.J. Research Institute of Chemistry, International Center for Chemical and Biological Sciences, University of Karachi, Karachi, Pakistan; 6 Dow Institute of Medical Technology, Dow University of Health Sciences, Karachi, Pakistan; University of Education, PAKISTAN

## Abstract

Charmagaz seed oil (CSO), derived from Cucurbitaceae family, and is a traditional mix of four different seeds: pumpkin, cucumber, watermelon, and musk melon. Widely utilized in Asia, this blend is considered as a brain tonic and a nutritional powerhouse. Despite its significant ethno medicinal value, the potential toxicity and safety of this combination oil have not been scientifically documented. Therefore, the current study was conducted to assess the sub-acute toxicity of Charmagaz seed oil in rat model for the assessment of its safety profile. For acute oral toxicity CSO was given at doses of 50, 500, and 5000 mg/kg orally over 28 days in rats. No evidence of toxicity was observed in animals when acutely exposed to CSO, implying that the LD50 is higher than 5000 mg/kg body weight. Cell viability assay revealed that Charmagaz oil is relatively non-toxic, showing an inhibition rate below 50% following 24 and 48-hour exposures. In the Brine Shrimp bioassay, oil demonstrated no cytotoxic effects, unlike the standard cytotoxic drug Etoposide, which resulted in 70% mortality at a 7.5 μg/ml concentration. No treatment-related toxicity or death was seen in any of the animals receiving dosages of 500 and 5000 mg/kg during the course of the 28-day study period, according to sub-acute oral toxicity tests. Additionally, no significant alterations (p > 0.05) were detected in hematological or biochemical parameters across the dosed groups. The administration of Charmagaz seed oil led to a modest elevation in high-density lipoprotein cholesterol, likely due to its polyunsaturated fatty acids content. Since the liver is a major organ involved in lipid metabolism hence, histopathological analysis was conducted to determine the effects of Charmagaz oil on hepatic tissues. The analysis revealed a positive correlation with biochemical results from liver function enzyme tests. Thus, this study provides evidence suggesting the safety of Charmagaz oil consumption at doses up to 5000 mg/kg.

## 1. Introduction

Subacute toxicity testing is essential for evaluating the reversibility of adverse effects, establishing dose-response relationships, and identifying target organ toxicity following repeated exposure in test animals. With the increasing utilization of natural extracts as therapeutic and functional agents, concerns regarding their safety have emerged [1]. Despite their popularity due to accessibility and affordability [2], many plant-based treatments lack sufficient toxicity data. Rigorous studies are needed to build confidence in their safe use [3]. With the WHO establishing guidelines and 80% of the population relying on herbal medicine, understanding potential toxic effects and informing consumers about risks is crucial [4]. The rising prevalence of herbal medicine use, driven by accessibility, affordability, and perceived safety, necessitates a thorough evaluation of potential toxicities. While traditional use suggests safety, limited toxicity data and recent reports of adverse effects raise concerns. Contamination by heavy metals, aflatoxins, and pathogenic microbes during preparation, as well as metal accumulation from soil, are potential sources of toxicity. Consequently, toxicity studies are crucial to establish the safety profile of medicinal plants and enhance consumer confidence [5].

The Cucurbitaceae family is known as guard & vine family, and includes around one thousands of species in about one hundred genus of plants. In human food conception, this family occupies the highest rank among the plant families and the major crop species of the family are Cucumber (*Cucumis sativus*), Pumpkin (*Cucurbita maxima),* Watermelon (*Citrullus lanatus*) and Musk melon (Cucumis melo) [6]. Plants within this family possess a long-standing history of ethno pharmacological and traditional medicinal use, supported by extensive documentation in traditional literature. The demonstrated phytochemical and pharmacological potential suggests these plants represent a promising source for the development of novel therapeutic compounds and functional food products [7]. The Cucurbitaceae family is established as a significant source of secondary metabolites exhibiting diverse biological activities, as evidenced by in vitro, in vivo, and clinical trial investigations [8].

Cucurbit seeds are significant in Islamic and Quranic contexts and are known for their traditional uses in Ayurveda and Chinese medicine [9]. Ethno medicinal literature indicates that these seeds are used to treat various health issues, including stomach disorders, intestinal parasites, dizziness, kidney inflammation, and depression [10]. Members of cucurbitaceae family have broad range of pharmacological activities including antioxidant [11,12], antitumor [13], antidiabetic [14], hepatoprotective [15], anti-obesity [16] and neurodegenerative diseases [17]. Numerous in-vitro and in-vivo investigations have linked the Cucurbita genus to these health-promoting properties. Clinical research results indicate that people with diabetes may benefit from cucurbita [18], patients with benign prostate hyperplasia, infertile women [19], postmenopausal women [20], and stress urinary incontinence in women [21,22].

Cucurbitaceae species are rich in diverse phytochemicals, including cucurbitacins, saponins, carotenoids, phytosterols, polyphenols, flavonoids, alkaloids, triterpenoids, and fatty acids, which contribute to their potential medicinal properties [23]. Seeds further contain cucurbitosides and lutein [23,24], and are a valuable source of lipids, proteins, carbohydrates, minerals (calcium, phosphorus, iron), and vitamin A [9]. The abundance of these bioactive compounds positions Cucurbitaceae as a promising source for novel natural therapeutics. Notably, Cucurbita species are abundant in natural antioxidants, such as polyphenols, terpenoids, and polyunsaturated fatty acids, which play a significant role in disease prevention. Phenolic compounds exhibit antioxidant activity through various mechanisms, including free radical scavenging, metal chelation, and modulation of enzyme activity, and may also enhance the action of other antioxidants [25].

Charmagaz is a traditional blend of four cucurbit seeds which are pumpkin (*C. maxima*), cucumber (*C. sativus*), watermelon (*C. lanatus*), and musk melon (*C. melo*). In literal terms, ‘char’ means four and magaz signify ‘brains’, i.e., four seeds that enhance cognitive abilities and increase memory. This combination is extensively used in Asia as a brain tonic and super food. The application of Cucurbitaceae members in public health, as nutraceuticals is associated with good ethno pharmacological proﬁle and easy availability [10]. To the best of our knowledge, no published article is found in literature search concerning the possible toxic effect of the combination of four cucurbits cold-pressed seed oil. Therefore, the present study was designed to evaluate the sub-acute toxicity and safety of Charmagaz seed oil in rat models.

## 2. Materials and methods

### 2.1. Ethical statement

All animal experiments were conducted in accordance with the National Institute of Health’s (NIH) guide for the care and use of laboratory animals. The protocol was reviewed and approved by the Institutional Bioethical Committee of University of Karachi under approval number (IBC KU-413/2024) and the Advanced Studies and Research Board (Reference No. 05918/Pharm). All efforts were made to minimize animal suffering and to reduce the number of animals used.

All staff received specialised training in animal care and handling to ensure compliance with NIH regulations.

### 2.2. Collection of the plant material

Cucurbit fruits were bought from the nearby local market from which the seeds were removed, shade dried and were deposited at Herbarium, Centre for Plant Conservation, University of Karachi. The taxonomist identified and issued the specimen number 94589 to Cucumis sativus, 9501 to Cucurbita maxima, 94494 to Cucumis melo and 9462 to Citrullus lanatus for reference.

### 2.3. Extraction of Charmagaz seed oil

Charmagaz seed oil was received as an extract of four cucurbit (*Cucumis sativus*, *Cucurbita maxima, Citrullus lanatus*, *Cucumis melo*) seeds by cold pressing using vegetable oil screw press model Komet DD 85 G (IBG MonfortsOekotec GmbH & Co. KG, Monchengladbach, Germany), Cold pressing is done three times with directly pressing the raw/dried seed by continuous screw press at low temperature. This technique entails an extraction of a solid sample oil contained within a plant hopper in a cold process. Oil extraction was performed to get the Cucurbitaceae seeds (of 0. 25 kg each of the studied varieties altogether 1 kg) ground and subjected to pressure exerted with the help of the conical screw rotation. The pressure was applied on the oil and it was compelled to enter into the perforated tube. After that at the end of the shaft, the prescribed quantity of meal was ejected by calibrated orifice which generally behaves as a barrier to the flow of meal that may contain residual fat or nutrition value. The remaining oil transacts to the centrifuge for 15 minutes in order to filter the oil with plant material particles. This is then followed by filtration [26]. After further weighing the extracted oils, the following formula was used to calculate the yield percentage:


$$\text{\textbackslash{}\% yield\textbackslash{} of\textbackslash{} oil}=\frac{\text{Weight\textbackslash{} of\textbackslash{} oil}}{\text{Weight\textbackslash{} of\textbackslash{} sample }(\text{g})}\times 100$$

### 2.4. Drugs and chemicals

Sigma Aldrich (MO, USA) provided the dimethyl sulfoxide (DMSO), methylthiazolyldiphenyl-tetrazolium bromide (MTT), and 2, 2-diphenyl-1-picrylhydrazyl (DPPH). The ATCC® CRL 1658 was used to obtain the viability of the NIH/3T3 cell line. Brine shrimp eggs (Artemia salina) were purchased from Ocean Nutrition CA, United States.

### 2.5. Determination of fatty acid composition by Gas chromatography flame ionization detection (GC-FID)

The analysis of fatty acid content was done in an Agilent 7890A gas chromatography equipped with a flame ionization detector (GC-FID) (Agilent Technologies, USA). The Charmagaz oil sample’s fatty acid methyl esters (FAMEs) were made using a 2N potassium hydroxide solution in methanol, following the procedure outlined by Akin et al. (2018). Briefly, 10 milliliters of hexane were used to dissolve 0.1 grams of Charmagaz seed oil that had been weighed into a centrifuge tube. A vigorous 60-second shaking was then applied to the tube along with 0.1 mL of 2N potassium hydroxide solution. Next, for five minutes at 2000 rpm, the mixture was centrifuged. Finally, the top layer was transferred to a vial for examination. We followed Gu et al. (2011) guidelines for operating conditions for FAME analysis, with a few minor adjustments [27]. Agilent’s HP-5MS (30m × 0.25 mm × 0.25µm) capillary column was used for the separations. Using a split ratio of 100:1, the injection volume was 1 µL. The temperature at the inflow was 230 °C. The temperature schedule for the oven was set to start at 60 °C and grow linearly to 175 °C (15 °C/min) and 240 °C (2 °C/min), maintaining these levels for ten minutes. Under the suggested procedure conditions, fatty acids were determined as g/100 g of total fatty acids by measuring the retention periods of a FAME mix C14:0–C22:0 standard that was administered. Using an Agilent ChemStation 2001–2010 data processor, the data was processed [27].

### 2.6. Antioxidant assay by DPPH method

A sample of Charmagaz oil was subjected for its capacity to scavenge the stable 2, 2-diphenyl-1-picrylhydrazyl (DPPH) compound. For this test, one milliliter of a reagent solution of 0. 5 grams of the oil dissolved in 5 mL of ethyl acetate was mixed vigorously with 4 mL of DPPH solution (prepared and diluted to 10 − 4 M ethyl acetate) in a screw capped test tube with about 10 mL capacity. The reaction mixture then stood for 10 seconds to allow for the formation of the emulsion using the Vortex apparatus. The tube was then left for 30 minutes in complete darkness as the system began to achieve steady state dark adaptation. Subsequently, the amount of the mixture was determined by absorbance at 515 nm against a reagent blank. At the same time, a control sample, which was constituted by combining 1 milliliters of ethyl acetate and 4 milliliters of DPPH solution, was prepared and measured as well. Percentage inhibition of DPPH radical formation (I %) was determined using the following formula and Methanol was used pertaining to this test as the control group [28].


I (%)=Absorbanceof\ control−Absorbance\ of\ sample/Absorbance\ of\ control×100

### 2.7. MTT assay using NIH/3T3 cell line

The MTT (Methylthiazolyldiphenyl-tetrazolium bromide) test was used to examine the cytotoxic potential of Charmagaz seed oil using the NIH/3T3 (ATCC® CRL 1658) cell line. The cells were cultured at 37°C in an incubator with 5 percent carbon-dioxide and maintained in DMEM supplemented with 10 percent fetal bovine serum. Every 24 hours, the medium was added in order to obtain 70–80% confluency. 6000 cells per well were collected and placed in 96-well plates with 200 µL of growth media in each well. A medium containing the test chemical at a concentration of 25–75 µM for Charmagaz seed oil was added after the 24-hour incubation period. Thus the untreated wells functioned as a control of positive growth. Each well was filled with 5 mg/ml MTT dye and incubated for an additional 3 hours. After removing the supernatant, 100 microliter of Dimethyl sulfoxide was introduced to each well in order to dissolve the formazan crystals. After 15 seconds of shaking the 96-well plate, the absorbance at 550 nanometer was measured using a spectrophotometer (Multiskan GO, Thermo Scientific). The following formula was used to calculate the percentage of cytotoxicity [29];


$$\%\text{ Cytotoxicity}=\frac{100-\text{O.D\textbackslash{} of\textbackslash{} treated\textbackslash{} well}-\text{O.D.\textbackslash{} of\textbackslash{} media\textbackslash{} control}}{\text{O.D\textbackslash{} of\textbackslash{} untreated\textbackslash{} control}-\text{O.D.\textbackslash{} of\textbackslash{} media\textbackslash{} control}}\;\times 100$$

### 2.8. Brine shrimp bioassay

Charmagaz seed oil was evaluated for its acute toxicity using a bioassay that is known as Brine shrimp (Artemia salina) method. Cold pressed seed oil was diluted in Dimethyl sulfoxide (DMSO) and the volume was made up to 5mL with sea water of different concentrations, i.e., 10, 100 and 1000 µg/mL. The test was done in replicates and control consisting only of Dimethyl sulfoxide in seawater. Eggs were sown in natural sea water and nauplii were obtained after 48h and used to inoculate the test samples [30]. After 24h of incubation at room temperature in the light, the number of survivors in each test tube was counted and the percentage mortality [31] was calculated by following formula:


% mortality=(test−control/control)×100

### 2.9. Sub-acute oral toxicity

In the present study, adult healthy Sprague Dawley rats (180–200 g, both sex) were purchased from the breeding unit of HEJ Institute of Chemistry, University of Karachi, Pakistan. The animals were housed in individual cages and were kept in standard settings, which included a 12:12 h light/dark cycle, a regulated room temperature of 23 ± 2 °C, stress-free, unlimited access to water, standard diets, and odor-free surroundings. The rats were given a week to become used to their new environment in order to minimize stress before the experiment began. We took all the necessary steps to reduce the number of animals used and their suffering.

#### 2.9.1. Experimental groups and treatment.

The rats were divided up into four groups, each with 10 animals (five males and five females). Group I served as vehicle control and was given purified water orally. The other three groups, referred to as groups II, III, and IV, were given oral doses of Charmagaz seed oil for a duration of 28 days, at dosages of 50, 500, and 5000 mg/kg respectively. After receiving Charmagaz seed oil, each animal was observed every day to look for any changes in their overall behavior or other physiological functions. Every animal was euthanized using ketamine at a dose of 100 mg/kg, followed by cervical dislocation to ensure death [32,33].

#### 2.9.2. Animal monitoring and welfare considerations.

Throughout the study, each animal was observed daily for any changes in overall behavior, physical appearance, and physiological functions, including mobility, grooming, feeding and excretory patterns. Monitoring was conducted at least twice daily to assess animal health, detect early signs of distress and identify severe adverse effects. Body weights were recorded weekly, and any signs of toxicity, such as lethargy, dehydration, abnormal breathing or convulsions were noted.

#### 2.9.3. Humane endpoints and euthanasia criteria.

Specific human endpoints were established to ensure that animals were euthanized if they exhibit signs of severe distress or if their condition was deteriorating beyond recovery. The humane endpoints included:

- Rapid weight loss exceeding 20% body weight
- Inability to eat or drink
- Persistent labored breathing or unresponsive behaviors
- Signs of severe pain or distress that could not be alleviated

Animals that reached any of these human endpoints were immediately euthanized using ketamine at a dose of 100 mg/kg followed by cervical dislocation to ensure death.

#### 2.9.4. Number of animals used and outcome.

A total of 40 animals were used in the study, with 10 animals in each group. No animals were found dead during the study period prior to euthanasia. All animals were euthanized at the end of the 28-day experimental period.

#### 2.9.5. Efforts to minimize suffering.

Throughout the experiment, every effort was made to minimize animals suffering. Animals were housed in comfortable, odor-free surroundings with minimal environmental stress. Additionally animals that displayed any signs of pain or distress were monitored closely, and any adverse symptoms were managed as per protocol. Analgesics or other supportive measures were considered, if necessary, though no analgesics were required during the course of this study.

#### 2.9.6. Duration of the experiment.

The experiment was conducted over 28 days, with daily observation and recording of health and behavior.

#### 2.9.7. Determination of hematological parameters.

White Blood Cells (WBCs), Red Blood Cells (RBCs), hemoglobin (Hb) and platelets (PLT) counts were assessed in blood samples using hematology analyzer (Siemens AG, Berlin, Germany).

#### 2.9.8. Determination of biochemical parameters.

High density lipoprotein cholesterol (HDL), total cholesterol (TC), Alanine Aminotransferase (ALT), Aspartate Aminotransferase (AST) and Creatinine (Cr) were performed by a local laboratory.

#### 2.9.9. Histopathological examination.

Livers were obtained and brought into the laboratory for immediate fixation in 10% phosphate buffered formalin, processed for paraffin embedding and 5 micrometer sections were made along the longitudinal axis. The sections were stained with hematoxylin and eosin (H&E) to observe under the microscope [34].

### 2.10. Statistical analysis

The data was presented as mean± S.E.M. Using SPSS version 23, a one-way ANOVA and Bonferroni’s post hoc test were used to assess the statistical significance of the group differences. Statistical significance was defined as values of p<0.05 and p̂ 0.001.

## 3. Results

### 3.1. Extraction and evaluation of lipid content

The cold-pressed process yielded a total percentage of 57.5% for the lipid content of Charmagaz seed oil. Cucurbitaceae seeds have a high total lipid content, which may make them economically advantageous for industrial extraction. This is especially true when compared to other oilseed crops like soybean and corn, which have lipid concentrations of 18–20% and 3.1–5.7%, respectively [35].

### 3.2. Charmagaz seed oil analysis by gas Chromatography Fame Ionization Detection (GC-FID)

An overview of Charmagaz seed oil’s fatty acid composition is shown in Table 1 (S1 Fig). Due to the inclusion of key fatty acids including oleic, palmitic, stearic, and linoleic, this oil falls within the category of oleic-linoleic acid [36]. Due to their high proportion of unsaturated fatty acids (UFAs, 76.662%) and low percentage of saturated fatty acids (SFAs, 23.338%), these fatty acid compositions are thought to be perfect for edible oils. The results showed that the polyunsaturated fatty acids (PUFAs) were 41.754% and the monounsaturated fatty acids (MUFAs) were 34.908%.

**Table 1:** Fatty acid profile of Charmagaz Seed oil by GC-FID.

Peak #	Name	RT (min)	Height	Area	Area%
1	C14:0 Myristic acid	37.112	0.91	2.22	0.816
2	C16:0 Palmitic acid	39.931	13.94	39.58	14.514
3	C18:0 Stearic acid	42.536	6.34	20.19	7.402
4	C18:1 Oleic acid	43.349	20.48	65.41	23.988
5	C18:2 Linoleic acid (Trans)	44.597	34.48	107.15	39.293
6	C18:2 Linoleic acid (cis)	44.877	1.58	4.40	1.612
7	C20:0 Eicosanoid acid	45.070	0.55	1.65	0.606
8	C18:3 n-6 Linolenic acid	45.318	0.73	2.31	0.849
9	C22:1 Erucic acid	49.189	5.76	29.78	10.92
			**Total**	**272.69**	**100.00**

### 3.3. Antioxidant Activity by DPPH Assay

Charmagaz seed oil, after 30 min of incubation quenched 31.1% of DPPH radicals which is comparable to that reported by Rezig et al., (2012) [28]. This indicates that cucurbit seed oil can neutralize free radicals, meaning they contain substances that can directly interact with and neutralize DPPH.

### 3.4. MTT assay using NIH/3T3 cell line

**Fig 1:**
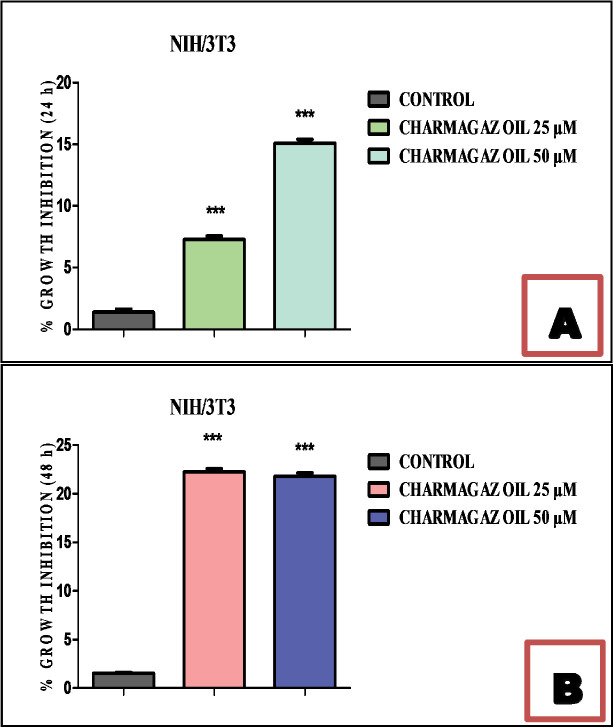
Effect of Charmagaz seed oil on normal NIH/3T3 fibroblast cell line using MTT assay. Three experiments were run and the data was represented as mean ± S.E.M. One-way ANOVA was used for data analysis. The significant values are labelled as ***p < 0.001, **p ≤ 0.01 and *p ≤ 0.05.

**Fig 2:**
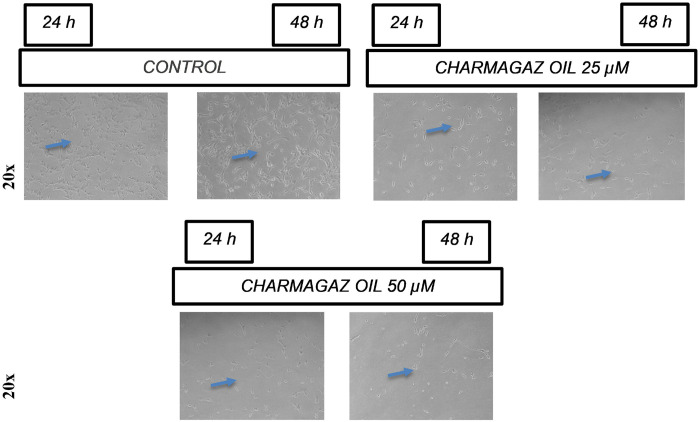
The images of the effect of Charmagaz oil produced after 24 h and 48 h treatment. The images demonstrate the effects of Charmagaz oil on fibroblast cells (NIH/3T3 cells). The arrows are used to show the effect produced by Charmagaz oil on 3T3 cells.

The cytotoxic potential of Charmagaz seed oil dissolved in DMSO) was observed using the NIH/3T3 cell line. This experiment was executed to screen for toxicity (if any) of Charmagaz seed oil on normal cells. The cytotoxicity profile was determined in terms of percent growth inhibition using MTT assay. Charmagaz oil was found considerably safe and showed less than 50% threshold of percentage inhibition after 24 h and 48 h treatment as shown in Fig 1. At the tested concentration of 25 µM and 50 µM, Charmagaz oil did not show any significant cytotoxic effect as all the cells were found viable. After 48 h, Charmagaz oil at concentration of 25 µM showed 22% killing. While other doses of Charmagaz oil after 24 h and 48 h showed less than 20% of growth inhibition. Thus, suggesting that our test oil is safe and harmless on cellular assay. The images are showing the very low toxic effects of Charmagaz oil (***p < 0.001) at 25 µM and 50 µM on NIH/3T3 cells after 24 h and 48 h treatment. Arrows are indicating the places where the cells are present with the normal morphology even after the treatment with Charmagaz oil as shown in Fig 2. However, after 48 h treatment with the test oil cells showing detachment but overall architecture of fibroblast cells were intact.

### 3.5. Brine Shrimp Bioassay

**Fig 3:**
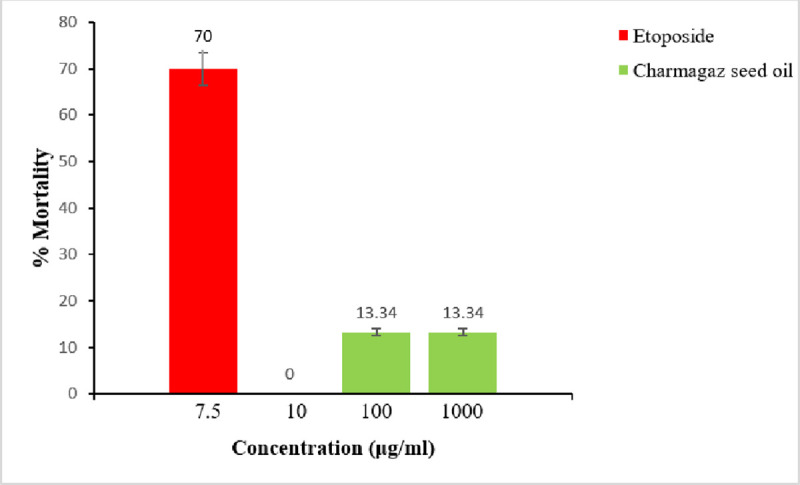
Percentage mortality of brine shrimps by Charmagaz seed oil.

As displayed in Fig 3 (S1 Table), Charmagaz seed oil exhibited no cytotoxic activity in relation to the standard drug Etoposide (70% mortality at 7.5 μg/ml concentration).

### 3.6. Acute oral toxicity

Oral administration of Charmagaz seed oil, at doses from 50 to 5000 mg/kg, did not produce any significant changes in behavioral responses (vocalization, self-trauma, isolation from cage mates, aggressiveness, ataxia, restlessness, grooming, hunched posture, porphyrin staining, soiled anogenital area etc.) and physiological responses (changes in body weight, water intake, food intake, body temperature, activity level, vital signs, respiratory distress etc.). During the 28-day experimental period, no deaths occurred in any of the groups. These results indicate that LD_50_ of Charmagaz seed oil is above 5000 mg per kg and the compound exerted substantial safety margin and seemed to be free from acute toxicity in rats.

### 3.7. Effect of Charmagaz seed oil on hematological parameters

As demonstrated in Table 2 (S2 Table), there was not a statistically significant difference in the relative blood parameters, such as RBCs and WBCs, between the Charmagaz seed oil-treated rats and the control group. Even at high dosages, Charmagaz seed oil does not seem to interfere with hematopoiesis, as shown by the lack of a discernible change in blood index levels in relation to the normal control group.

**Table 2:** Effect of Charmagaz seed oil on Hematological Parameters.

Parameters	Control	50 mg/kg	500 mg/kg	5000 mg/kg	P-value
RBCs (x 10^6^/µl)	8.74 ± 0.04	8.78 ± 0.03	8.69 ± 0.05	8.91 ± 0.09	0.106
WBCs (x 10^3^/µl)	9.6 ± 0.09	10.7 ± 0.11	10.6 ± 0.19	9.2 ± 0.12	0.000
Hb (g/dl)	15.7 ± 0.63	15.6 ± 0.11	16.0 ± 0.17	15.4 ± 0.15	0.652
Platelets (x 10^6^/µl)	746 ± 17.5	768 ± 25.9	750 ± 44.8	806 ± 19.3	0.465

Values expressed as Mean ± SEM, one-way ANOVA followed by Bonferroni’s test (n = 10). Differences between groups were signiﬁcant when p < 0.05. Red Blood Cells (RBCs); White Blood cells (WBCs) and Hemoglobin (Hb).

### 3.8. Effect of Charmagaz seed oil on biochemical parameters

The serum concentrations of Creatinine, AST and ALT were not affected significantly after the treatment with Charmagaz seed oil indicating no change in the normal level of these serum markers as shown in Table 3 (S3 Table), Furthermore, the value of the Total cholesterol and HDL was not reduced in the treated groups compared to the control group.

**Table 3:** Effect of Charmagaz seed oil on biochemical parameters.

Parameters	Control	50 mg/kg	500 mg/kg	5000 mg/kg	P-value
Creatinine (mg/dl)	0.61 ± 0.03	0.64 ± 0.03	0.67 ± 0.03	0.64 ± 0.03	0.656
ALT (IU/L)	96.9 ± 0.76	108.1 ± 0.69	113.1 ± 0.93	117.4 ± 0.85	0.000
AST (IU/L)	100.2 ± 2.1	120.7 ± 2.2	132.9 ± 3.9	176.1 ± 2.4	0.000
Total cholesterol (mg/dl)	72.1 ± 0.76	76.7 ± 2.2	76.3 ± 1.8	78.3 ± 1.6	0.086
HDL (mg/dl)	43.3 ± 0.11	44.3 ± 0.04	45.4 ± 0.18	45.3 ± 0.68	0.001

Values expressed as Mean ± SEM, one-way ANOVA followed by Bonferroni’s test (n = 10). Differences between groups were signiﬁcant when p < 0.05. ALT = Alanine Aminotransferase, AST = Aspartate Aminotransferase and HDL = high density lipoprotein cholesterol.

### 3.9. Effect of Charmagaz seed oil on histopathology

**Fig 4:**
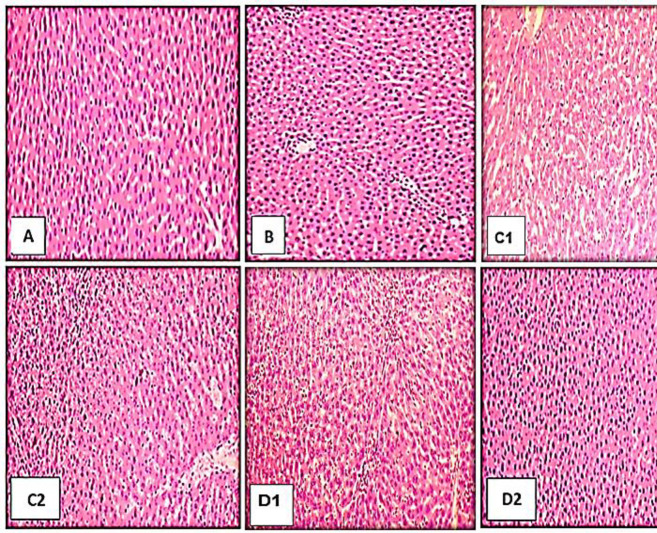
Photometry of liver of control group (A), group treated with 50 mg/kg (B), group treated with 500 mg/kg (C1, C2) and group treated with 5000 mg/kg (D1, D2). Histopathological assessment of liver of Charmagaz seed oil treated groups showed no pathological changes after 28 days treatment as compared to control group.

According to Fig 4, histological analysis of the liver tissue in the normal control group (A) demonstrated normal parenchyma with intact hepatic architecture. Hepatic tissue of 50 mg/kg group (B) of Charmagaz seed oil revealed normal parenchyma with intact architecture. Hepatic tissue of animals at the doses of 500 mg/kg (C1, C2) displayed an intact cellular architecture. Likewise, photomicrographs of hepatic tissue treated with 5000 mg/kg (D1, D2) revealed intact architecture devoid of any signs of necrosis, edema, or degenerative alterations.

## 4. Discussion

Plant-based herbal medicines have minimal adverse effects making them typically safe and effective. The rural populace may have used herbal medicines responsibly because they were thought to be safe. In developing countries with large rural populations, these preparations are often taken orally for extended periods without proper consultation or dosage control, which can lead to a lack of awareness about potential toxic effects. Consequently, there is a need for scientifically validated information on the oral toxicity of herbal medicines. Such data would not only assist in identifying safe dosages for future use but also reveal various clinical signs and symptoms related to the herbal agent being studied. While the pharmacological effects of all the four members from Cucurbitaceae family have been established, comprehensive knowledge about the toxicity of combination preparation is still missing. Therefore, this research study was conducted to assess the safety data of Charmagaz seed oil [37,38].

Toxicological characteristics of the plant extract or isolated components are examined as the first stage in the pharmacological activity screening process. When studying new compounds in animal research, the acute toxicity profile offers information that is helpful for labeling, classifying, and determining the dosage. No treatment-related fatalities occurred over the duration of the present investigation, indicating that Charmagaz seed oil at a high dosage of 5000 mg/kg BW was neither toxic nor deadly. As every animal used in the research lived until they were compelled to be put to death, a lethal dosage (LD_50_) of more than 5000 mg/kg BW of Charmagaz seed oil was discovered in rats. To ensure that there were no observable adverse effect levels (NOAELs) in rats, three distinct doses—50, 500, and 5000 mg/kgwere chosen for the 28-day repeated dose toxicity trial, which was designed based on the LD_50_ dosage. The preclinical research on the acute toxicity of Charmagaz seed oil for clinical medication safety has not yet been found, as far as we could find. Because of this, we carried out a thorough pre-study for the present study in order to evaluate the safety and harmful effects of Charmagaz seed oil on rats. In another study, Atmane et al. (2022) investigated the safety profile of cold-pressed oil extracted from Pinus halepensis Mill. Seeds (COPHS) in mice. Their findings demonstrated that oral administration of COPHS at doses up to 5000 mg/kg body weight did not result in mortality or any observable signs of toxicity. This study suggests a large safety margin (>5000 mg/kg) for cold-pressed seed oils, providing some context for the potential safety of Charmagaz cold-pressed seed oil, though further research is certainly necessary [39]. It is important to note that while this provides a relevant comparison, it does not directly address the safety of Charmagaz oil, and our research aims to specifically address this gap. Therefore, we have retained and emphasized the importance of future toxicological studies on Charmagaz seed oil.

GCFID analysis of the seed oil were carried out using standard biochemical procedures that reveals the seed oils of Cucurbitaceae contain palmitic acid, Linoleic acid, Oleic acid, myristic acid and stearic acid that are 16–18 carbon fatty acids requires for normal growth, development and life processes.. Our findings are in accordance with Rezig et al., 2021 that reported Cucurbitaceae seeds presented an alternative source of plant oil as rich in omega 3 and 6 fatty acids which may serve as raw material for food applications and provide health benefits [40].

Cucurbita species plants are abundant in phytochemicals and serve as a rich supply of antioxidants. The phytochemicals Cucurbitacins, saponins, carotenoids, phytosterols, and polyphenols are the most significant ones found in cucurbits. Phenolic chemicals function in various ways, such as “scavenging” free radicals, and are members of a large class of antioxidants. The human body has several mechanisms to counteract oxidative stress by using antioxidants, which are either naturally produced in situ, or externally supplied through food and/or supplements. Endogenous and exogenous antioxidants interfere with the oxidation process by reacting with free radicals, chelating free catalytic metals, and acting as oxygen scavengers. Therefore, the consumption of cucurbits, which contain polyphenols and terpenoids in a large amount, plays an important antioxidant role in prophylaxis against oxidative stress [41].

The DPPH test shows that Charmagaz seed oil has antioxidant activity. As reported by several studies, seeds concentrated a higher proportion of vegetable constituents like poly unsaturated fatty acids (PUFAs) which are both of nutritional or medicinal interest [41–43].

Similar to the findings reported by Rezig et al. (2012), 31.1% of DPPH radicals were quenched by cucurbit seed oil after 30 minutes of incubation [28]. Strong naturally occurring antioxidants including ascorbic acid, β-carotene, lycopene, and tocopherols are responsible for the free radical scavenging action. Our findings suggest that Charmagaz seed oil’s strong antioxidant levels may be a factor in its therapeutic potential because they may provide protection against oxidative damage brought on by free radical scavenging.

In the MTT assay, Charmagaz oil was determined to be relatively safe, demonstrating less than a 50% inhibition rate after treatments of 24 and 48 hours, as shown in Fig 1. Similarly, in Brine Shrimp (Artemia salina) bioassay, Charmagaz seed oil did not exhibit cytotoxic effects when compared to the standard drug Etoposide, which caused 70% mortality at a concentration level of 7.5 microgram per ml as shown in Fig 3. Acute toxicity research further supported the safety of cucurbit seed oil. Acute oral toxicity tests identify obvious unfavorable effects that may impair the lifetime of the experimental animals and give crucial information on dosing schedules and target organ toxicity. In the present investigation, the effect of Charmagaz seed oil was evaluated in rats at doses of 50, 500 and 5000 mg/kg for 28 days. It has been observed that acute administration of Charmagaz seed oil did not produce any significant changes in behavioral and physiological responses. During the 28-day experimental period, no deaths occurred in any of the groups. Our findings agree with the previously reported safety profile of Cucurbita maxima and Cucumis sativus by Wahid et al., (2020) and Cruz et al., 2006 respectively [44,45]. The acute oral toxicity study showed that the LD_50_ of Charmagaz seed oil exceeds 5000 mg/kg. This suggests a significant safety margin, as they appear to be free from sub-acute toxicity in rats.

The hematopoietic system is the most susceptible system for toxic substances and becomes a useful marker of physiological and pathological state in animals and humans. Being the major medium for transporting nutrient and other foreign substances, the hematological parameter can be useful in evaluating the degree of harm caused by foreign compounds. In the present research study, all these hematological parameters were found to be within reference range of rat, indicating that Charmagaz seed oil does not contain any toxic compounds which could cause any variation in normal physiology. Charmagaz seed oil showed no significant changes on hematological variables recorded between the rats in the experimental group and the control group. The outcomes have been presented in tabular form in Table 2. The seed oil was also found to be inactive at a dose of 5000 mg per kg on synthesis and proliferation of blood cells or destruction at any stage.

Biochemical parameters are very essential in safety evaluation as they act as indicators in response to signs and symptoms elicited by toxicants. Therefore, to evaluate the toxic effects attained by seed oil, determination of liver and kidney function is mandatory. It is important to comprehend that the liver is the second largest internal organ in the human body. Extremely it can be damaged by several toxic molecules and medicinal agents taken in overdoses. This is explained by the fact that damage to the liver cell’s plasma membrane releases elevated amounts of liver function enzymes into the circulation. Aspartate transaminase and Alanine aminotransferase are predominantly located in the hepatic cells, and rise in their concentrations reflect the injury to the said cells. As a result, studying serum concentrations of ALT and AST can be regarded as the most informative markers of hepatocyte damage [46]. In the current study, no changes were found in the levels of Alanine aminotransferase and Aspartate aminotransferase thus implying that oil has hepatoprotective action as depicted in Table 3 [47]. In a similar way, the obtained result support the study carried out by Abou Seif (2014) and Bazabang et al. (2018) [48,49] which revealed that Cucurbita maxima seed oil and Citrullus lanatus seed extract possess hepatoprotective effect in alcohol induced hepatotoxicity and oxidative stress. Furthermore, no histological alterations of the liver cells were evidenced in the treated animals at all the doses (the low, middle, and high doses), proposing the hepatoprotective effect of cucurbit oil on hepatic tissues. In this study, we have got a positive correlation between liver function test and histological architecture of liver tissues.

Liver is a crucial organ for lipid metabolism as it secretes bile which helps in the absorption of fats and fat-soluble vitamins. The effect of Charmagaz seed oil on lipid metabolism is an important parameter to determine its safety as the oil itself is metabolized by liver. Charmagaz seed oil belong to the oleic-linoleic acid type of oil owing to the presence of major fatty acids such as Linoleic, oleic, palmitic, and stearic (Table 1). These fatty acids are regarded as the first choice of edible oils because they contain a high percentage of unsaturated fatty acids and low percentage of saturated fatty acids [36]. Dyslipidemia is characterized by an abnormal blood lipid profile, is usually diagnosed from Triglycerides (TGs), Total Cholesterol (TC), Low density lipoprotein cholesterol (LDL-C), and High-density lipoprotein cholesterol (HDL-C) levels [50]. Interestingly, Charmagaz seed oil decreased total cholesterol and increased the levels of High-density lipoprotein cholesterol indicating its usefulness in the management of hypercholesterolemia (Table 3). Moreover, cucurbit oil at any of the dose did not derange the values of liver enzymes and lipid profile as compared to normal control indicating hepatoprotective activity which is due to the presence of PUFAs such as palmitic acid, linoleic acid, oleic acid, and stearic acid [51]. Moreover, Poly unsaturated fatty acid (PUFAs) including linoleic acid (LA) and alpha-linolenic acid (ALA) have shown antioxidant property by preventing lipid peroxidation and raising the levels of many cellular antioxidants including ascorbic acid, α-tocopherol and glutathione [52].

Serum creatinine concentration is the surrogate test used to determine renal function. Importantly, Serum creatinine is acknowledged as an inadequate gold standard for several reasons. Besides Liver, Kidney is also a very important organ in the body that helps to regulate the whole body by throwing out metabolism waste materials. Long term consumption of oil might harm the renal tubules of kidneys and thus produce nephrotoxicity. In order to evaluate normal kidney function, we assessed the degree of normal kidney function through serum creatinine levels after administering oil for 28 days. Serum creatinine concentration which tends to increase in cases of impaired kidney function was unaltered in the treated rats in relation to normal control as shown in (Table 3).

Along with the predominant PUFAs, cucurbitaceae seeds also possess high content of polyphenols (protocatechuic, p-coumaric, vanillic), squalene, tocopherols, phytosterols, and carotenoids (lycopene, lutein, and zeaxanthin). These phytoconstituents show a positive correlation with the plant’s nephron-protective activity [53,54].

Histopathological examination of hepatic tissues in the control group as shown in Fig 4 (A) demonstrates typical architecture of hepatocytes indicated by centrally & rounded nuclear membrane with clear cellular boundary. While evaluation of liver tissues of treated groups showed that Charmagaz seed oil did not affect the normal architecture compared to normal control. Hepatic tissue of animals at the doses of 500 mg/kg (C1, C2) and 5000 mg/kg (D1, D2) showed intact architecture. Also, no evidence of degenerative changes, edema or necrosis was noted. Our findings are in support of Belemkar and Shendge, 2021 who reported the protective effect of Citrullus lanatus seed extract on histopathology of liver tissues in rats. Moreover, the biochemical results (ALT, AST) further strengthened the histopathological findings as they interpret the normal liver function [55].

While traditional medicine systems and modern pharmaceuticals offer valuable approaches to healthcare, incorporating nutrient-rich plant-based foods, like cucurbit seeds, can play a vital role in disease prevention and health promotion [56]. Cucurbitaceae seeds are a promising source of bioactive compounds and can be utilized in functional foods [57]. These seeds are rich in beneficial compounds [58] and offer a sustainable dietary option due to their adaptability [57]. However, despite their nutritional value, cucurbit seeds, particularly from pumpkin and watermelon, are often underutilized and wasted [59]. Given the increasing focus on plant-based diets [58] and the challenges of food security [59], maximizing the use of these nutrient-dense seeds in diverse food applications, including nutraceuticals, represents a significant opportunity to improve community health and potentially lessen reliance on pharmaceutical interventions.

## 5. Conclusion

The current research study indicates that an oral dosage of up to 5000 mg/kg of Charmagaz seed oil is considered safe, as it did not exhibit any signs of sub-acute toxicity or cause mortality in rats. Furthermore, the oil did not result in any negative effects on the normal behavior of treated animals. During a 28-day period of repeated administration, the biochemical, hematological, behavioral, and histological indicators remained within the safe range for dosages of 50, 500, and 5000 mg/kg, in comparison to the untreated control group. These findings suggest that Charmagaz seed oil can be safely utilized in the evaluation of pharmacological and therapeutic effects. Moreover, these preclinical results also provide a basis to further explore the efficacy of Charmagaz seed oil in clinical practice. While the demonstrated benefits of cucurbit seeds are promising, further research, including detailed chronic and sub-chronic toxicity studies, is crucial to definitively establish their safety for widespread use. Additional investigation into the medicinal and therapeutic efficacy is also warranted to fully understand and leverage the potential health benefits of Cucurbitaceae seed oils.

## Supporting information

S1 Fig Fatty Acid Profile of Charmagaz Seed Oil by GC-FID. (PDF)

S1 Table Percentage mortality of brine shrimps by Charmagaz seed oil. (DOCX)

S2 Table Effect of Charmagaz seed oil on Hematological Parameters. (XLSX)

S3 Table Effect of Charmagaz seed oil on Biochemical Parameters. (XLSX)

## References

[1] Li Y, Zhuang Y, Tian W, Sun L. In vivo acute and subacute toxicities of phenolic extract from rambutan (Nephelium lappaceum) peels by oral administration. Food Chem. 2020;320:126618. doi: 10.1016/j.foodchem.2020.126618 32229397

[2] Ahmed RK, Syarhabilahmad M, Nabil M, Al–Suede FSR. Acute and sub-chronic toxicity study of encapsulation of combine plants extract of Ficus deltoidea and Gynocthodes sublanceolata in Balb/c mice model. J Angiothe. 2019;3:147–55.

[3] Saggar S, Mir PA, Kumar N, Chawla A, Uppal J, Kaur A. Traditional and herbal medicines: opportunities and challenges. Pharmacognosy Research. 2022;14(2).

[4] Başaran N, Paslı D, Başaran AA. Unpredictable adverse effects of herbal products. Food. Chemical Toxicology. Chem Toxicol. 2022;159:112762. 10.1016/j.fct.2021.112762 34896186

[5] Shendge PN, Belemkar S. Acute and 28-day oral toxicity studies of methanolic extract of Lagenaria siceraria (Cucurbitaceae) fruit in rats. Drug Chem Toxicol. 2021;44(5):493–501. doi: 10.1080/01480545.2019.1617302 31146591

[6] Jamuna S, Karthika K, Paulsamy S. Phytochemical and pharmacological properties of certain medicinally important species of Cucurbitaceae family—A review. Res Biol. 2015;6:1835–49.

[7] Mukherjee PK, Singha S, Kar A, Chanda J, Banerjee S, Dasgupta B, et al. Therapeutic importance of Cucurbitaceae: A medicinally important family. J Ethnopharmacol. 2022;282:114599. doi: 10.1016/j.jep.2021.114599 34487849

[8] Huerta-Reyes M, Tavera-Hernández R, Alvarado-Sansininea JJ, Jiménez-Estrada M. Selected Species of the Cucurbitaceae Family Used in Mexico for the Treatment of Diabetes Mellitus. Molecules. 2022;27(11):3440. doi: 10.3390/molecules27113440 35684376 PMC9182361

[9] Nain P, Kumar S, Bhatia M, Kaur J. Enhancing cognitive performance with rejuvenation of brain antioxidant markers and acetylcholinesterase activity by ethanolic extract of Cucurbita pepo L. seeds in scopolamine-induced model of dementia in rats. Journal of Reports in Pharmaceutical Sciences. 2021;10(2):271–8.

[10] Salehi B, Capanoglu E, Adrar N, Catalkaya G, Shaheen S, Jaffer M, et al. Cucurbits Plants: A Key Emphasis to Its Pharmacological Potential. Molecules. 2019;24(10):1854. doi: 10.3390/molecules24101854 31091784 PMC6572650

[11] Beshay EVN, Rady AA, Afifi AF, Mohamed AH. Schistosomicidal, antifibrotic and antioxidant effects of Cucurbita pepo L. seed oil and praziquantel combined treatment for Schistosoma mansoni infection in a mouse model. J Helminthol. 2019;93(3):286–94. doi: 10.1017/S0022149X18000317 29655377

[12] Kostecka-Gugała A, Kruczek M, Ledwożyw-Smoleń I, Kaszycki P. Antioxidants and Health-Beneficial Nutrients in Fruits of Eighteen Cucurbita Cultivars: Analysis of Diversity and Dietary Implications. Molecules. 2020;25(8):1792. doi: 10.3390/molecules25081792 32295156 PMC7221643

[13] Ríos JL, Andújar I, Escandell JM, Giner RM, Recio MC. Cucurbitacins as inducers of cell death and a rich source of potential anticancer compounds. Curr Pharm Des. 2012;18(12):1663–76. doi: 10.2174/138161212799958549 22443631

[14] Acosta-Patiño JL, Jiménez-Balderas E, Juárez-Oropeza MA, Díaz-Zagoya JC. Hypoglycemic action of Cucurbita ficifolia on Type 2 diabetic patients with moderately high blood glucose levels. J Ethnopharmacol. 2001;77(1):99–101. doi: 10.1016/s0378-8741(01)00272-0 11483384

[15] Perez Gutierrez RM. Review of Cucurbita pepo (pumpkin) its phytochemistry and pharmacology. Med Chem. 2016;6(1):12–21.

[16] Kalaivani A, Sathibabu Uddandrao VV, Brahmanaidu P, Saravanan G, Nivedha PR, Tamilmani P, et al. Anti obese potential of Cucurbita maxima seeds oil: effect on lipid profile and histoarchitecture in high fat diet induced obese rats. Nat Prod Res. 2018;32(24):2950–3. doi: 10.1080/14786419.2017.1389939 29047298

[17] Di Domenico F, Coccia R, Butterfield DA, Perluigi M. Circulating biomarkers of protein oxidation for Alzheimer disease: expectations within limits. Biochim Biophys Acta Proteins Proteomics. 2011;1814(12):1785–95. 10.1016/j.bbapap.2011.10.001 22019699

[18] Jain A, Mishra M, Yadav D, Khatarker D, Jadaun P, Tiwari A. Evaluation of the antihyperglycemic, antilipidemic and antioxidant potential of Cucurbita ficifolia in human type 2 diabetes. Prog Nutr. 2018;20:191–8.

[19] Ushiroyama T, Yokoyama N, Hakukawa M, Sakuma K, Ichikawa F, Yoshida S. Clinical efficacy of macrophage-activating Chinese mixed herbs (MACH) in improvement of embryo qualities in women with long-term infertility of unknown etiology. Am J Chin Med. 2012;40(1):1–10. doi: 10.1142/S0192415X12500012 22298444

[20] Gossell-Williams M, Hyde C, Hunter T, Simms-Stewart D, Fletcher H, McGrowder D, et al. Improvement in HDL cholesterol in postmenopausal women supplemented with pumpkin seed oil: pilot study. Climacteric. 2011;14(5):558–64. doi: 10.3109/13697137.2011.563882 21545273

[21] Gažová A, Valášková S, Žufková V, Castejon AM, Kyselovič J. Clinical study of effectiveness and safety of CELcomplex® containing Cucurbita Pepo Seed extract and Flax and Casuarina on stress urinary incontinence in women. Journal of Traditional Complementary Medicine. 2019;9(2):138–42. 30963048 10.1016/j.jtcme.2017.10.005 PMC6435946

[22] Salehi B, Quispe C, Sharifi-Rad J, Giri L, Suyal R, Jugran AK, et al. Antioxidant potential of family Cucurbitaceae with special emphasis on Cucurbita genus: A key to alleviate oxidative stress-mediated disorders. Phytother Res. 2021;35(7):3533–57. doi: 10.1002/ptr.7045 33590924

[23] Rajasree R, Sibi P, Francis F, William H. Phytochemicals of Cucurbitaceae family—A review. International J Phytochem Res. 2016;8(1):113–23.

[24] Ratnam N, Naijibullah M, Ibrahim M. A review on Cucurbita pepo. Int J Pharm Phytochem Res. 2017;9:1190–4.

[25] Nawirska-Olszańska A, Kita A, Biesiada A, Sokół-Łętowska A, Kucharska AZ. Characteristics of antioxidant activity and composition of pumpkin seed oils in 12 cultivars. Food Chem. 2013;139(1–4):155–61. doi: 10.1016/j.foodchem.2013.02.009 23561092

[26] Rezig L, Chouaibi M, Meddeb W, Msaada K, Hamdi S. Chemical composition and bioactive compounds of Cucurbitaceae seeds: Potential sources for new trends of plant oils. Environ Protect. 2019;127:73–81.

[27] Gu Q, David F, Lynen F, Vanormelingen P, Vyverman W, Rumpel K, et al. Evaluation of ionic liquid stationary phases for one dimensional gas chromatography-mass spectrometry and comprehensive two dimensional gas chromatographic analyses of fatty acids in marine biota. J Chromatogr A. 2011;1218(20):3056–63. doi: 10.1016/j.chroma.2011.03.011 21450294

[28] Rezig L, Chouaibi M, Msaada K, Hamdi S. Chemical composition and profile characterisation of pumpkin (Cucurbita maxima) seed oil. Industrial Crops and Products. 2012;37(1):82–7. doi: 10.1016/j.indcrop.2011.12.004

[29] Molavi O, Torkzaban F, Jafari S, Asnaashari S, Asgharian P. Chemical compositions and anti-proliferative activity of the aerial parts and rhizomes of squirting cucumber, Cucurbitaceae. Jundishapur Journal of Natural Pharmaceutical Products. 2020;15(1).

[30] Atolani O, Omere J, Otuechere CA, Adewuyi A. Antioxidant and cytotoxicity effects of seed oils from edible fruits. Journal of Acute Disease. 2012;1(2):130–4. doi: 10.1016/s2221-6189(13)60030-x

[31] Doshi GM, Kanade PP. Evaluation of bioactivity of Cucurbita pepo L., Cucumis melo L. and Cucumis sativus L. Seed Extracts. 2019.

[32] Tobar Leitão SA, Soares DDS, Carvas Junior N, Zimmer R, Ludwig NF, Andrades M. Study of anesthetics for euthanasia in rats and mice: A systematic review and meta-analysis on the impact upon biological outcomes (SAFE-RM). Life Sci. 2021;284:119916. doi: 10.1016/j.lfs.2021.119916 34480936

[33] Jonsson M, Jestoi M, Nathanail AV, Kokkonen U-M, Anttila M, Koivisto P, et al. Application of OECD Guideline 423 in assessing the acute oral toxicity of moniliformin. Food Chem Toxicol. 2013;53:27–32. doi: 10.1016/j.fct.2012.11.023 23201451

[34] Calil Brondani J, Reginato FZ, da Silva Brum E, de Souza Vencato M, Lima Lhamas C, Viana C, et al. Evaluation of acute and subacute toxicity of hydroethanolic extract of Dolichandra unguis-cati L. leaves in rats. J Ethnopharmacol. 2017;202:147–53. doi: 10.1016/j.jep.2017.03.011 28288826

[35] Jorge N, da Silva AC, Malacrida CR. Physicochemical characterisation and radical-scavenging activity of Cucurbitaceae seed oils. Nat Prod Res. 2015;29(24):2313–7. doi: 10.1080/14786419.2015.1007135 25697079

[36] Akın G, Arslan FN, Elmas Karuk ŞN, Yılmaz İ. Cold-pressed pumpkin seed (Cucurbita pepo L.) oils from the central Anatolia region of Turkey: characterization of phytosterols, squalene, tocols, phenolic acids, carotenoids and fatty acid bioactive compounds. 2018.

[37] Féres CAO, Madalosso RC, Rocha OA, Leite JPV, Guimarães TMDP, Toledo VPP, et al. Acute and chronic toxicological studies of Dimorphandra mollis in experimental animals. J Ethnopharmacol. 2006;108(3):450–6. doi: 10.1016/j.jep.2006.06.002 16872769

[38] Yuet Ping K, Darah I, Chen Y, Sreeramanan S, Sasidharan S. Acute and subchronic toxicity study of Euphorbia hirta L. methanol extract in rats. Biomed Res Int. 2013;2013:182064. doi: 10.1155/2013/182064 24386634 PMC3872372

[39] Ait Atmane S, Ait Eldjoudi D, Aksoylu Özbek Z, Günç Ergönül P, Khettal B. Acute and 28-day repeated dose toxicity evaluations of cold pressed Pinus halepensis Mill. seed oil in mice and rats. Regul Toxicol Pharmacol. 2022;132:105191. doi: 10.1016/j.yrtph.2022.105191 35613671

[40] Rezig L, Chouaibi M, Meddeb W, Msaada K, Hamdi S. Chemical composition and bioactive compounds of Cucurbitaceae seeds: Potential sources for new trends of plant oils. Process Safety and Environmental Protection. 2019;127:73–81. doi: 10.1016/j.psep.2019.05.005

[41] Peña M, Guzmán A, Mesas C, Porres JM, Martínez R, Bermúdez F, et al. Evaluation of the Leaves and Seeds of Cucurbitaceae Plants as a New Source of Bioactive Compounds for Colorectal Cancer Prevention and Treatment. Nutrients. 2024;16(23):4233. doi: 10.3390/nu16234233 39683626 PMC11644257

[42] Kulczyński B, Sidor A, Gramza-Michałowska A. Antioxidant potential of phytochemicals in pumpkin varieties belonging to Cucurbita moschata and Cucurbita pepo species. CyTA-Journal of Food. 2020;18(1):472–84.

[43] Islam F, Imran A, Nosheen F, Fatima M, Arshad MU, Afzaal M, et al. Functional roles and novel tools for improving-oxidative stability of polyunsaturated fatty acids: A comprehensive review. Food Sci Nutr. 2023;11(6):2471–82. doi: 10.1002/fsn3.3272 37324849 PMC10261796

[44] Cruz R, Meurer C, Silva E, Schaefer C, Santos A, Bella Cruz A. Toxicity evaluation of Cucurbita maxima seed extract in mice. Pharmaceutical Biology. 2006;44(4):301–3.

[45] Wahid S, Alqahtani A, Alam Khan R. Analgesic and anti-inflammatory effects and safety profile of Cucurbita maxima and Cucumis sativus seeds. Saudi J Biol Sci. 2021;28(8):4334–41. doi: 10.1016/j.sjbs.2021.04.020 34354417 PMC8325025

[46] Alsahli MA, Almatroodi SA, Almatroudi A, Khan AA, Anwar S, Almutary AG, et al. 6‐Gingerol, a major ingredient of ginger attenuates diethylnitrosamine‐induced liver injury in rats through the modulation of oxidative stress and anti‐inflammatory activity. Mediators of Inflammation. 2021;2021(1):6661937. 33531877 10.1155/2021/6661937 PMC7837795

[47] Singh D, Cho WC, Upadhyay G. Drug-Induced Liver Toxicity and Prevention by Herbal Antioxidants: An Overview. Front Physiol. 2016;6:363. doi: 10.3389/fphys.2015.00363 26858648 PMC4726750

[48] Abou Seif HS. Ameliorative effect of pumpkin oil (Cucurbita pepo L.) against alcohol-induced hepatotoxicity and oxidative stress in albino rats. Appl Sci. 2014;3(3):178–85.

[49] Monday N, Bazabang S, Adebisi S, Makena W, Iliya I. Hepatoprotective Effects of Aqueous Extract of Watermelon (Citrullus lanatus) Seeds on Ethanol-Induced Oxidative Damage in Wister Rats. Sub-Saharan Afr J Med. 2018;5(4):129. doi: 10.4103/ssajm.ssajm_18_18

[50] Zhang Y, Wang Z, Jin G, Yang X, Zhou H. Regulating dyslipidemia effect of polysaccharides from Pleurotus ostreatus on fat-emulsion-induced hyperlipidemia rats. Int J Biol Macromol. 2017;101:107–16. doi: 10.1016/j.ijbiomac.2017.03.084 28322967

[51] Daoudi NE, Bouhrim M, Bnouham M. Review on Hepatoprotective Effects of Some Medicinal Plant Oils. Discovery. 2021;18(3):239–48.

[52] Nagao T, Komine Y, Soga S, Meguro S, Hase T, Tanaka Y, et al. Ingestion of a tea rich in catechins leads to a reduction in body fat and malondialdehyde-modified LDL in men. Am J Clin Nutr. 2005;81(1):122–9. doi: 10.1093/ajcn/81.1.122 15640470

[53] Negi K, Mirza A. Nephroprotective and Therapeutic Potential of Traditional Medicinal Plants in Renal Diseases. Journal of Drug Research in Ayurvedic Sciences. 2020;5(3):175–83. doi: 10.5005/jdras-10059-0079

[54] Oyetayo FL, Akomolafe SF, Osesanmi TJ. Effect of dietary inclusion of pumpkin (Cucurbita pepo L) seed on nephrotoxicity occasioned by cisplatin in experimental rats. J Food Biochem. 2020;44(10):e13439. doi: 10.1111/jfbc.13439 32808341

[55] Belemkar S, Shendge PN. Toxicity profiling of the ethanolic extract of Citrullus lanatus seed in rats: behavioral, biochemical and histopathological aspects. Biosci Rep. 2021;41(1):BSR20202345. doi: 10.1042/BSR20202345 33289840 PMC7796193

[56] Pattanayak S. Therapeutic use of plant parts as food or medicine at succulent state: an alternative healthcare system for the prevention and treatment of diseases. Discover Plants. 2025;2(1):35.

[57] Romo-Tovar J, Belmares Cerda R, Chávez-González ML, Rodríguez-Jasso RM, Lozano-Sepulveda SA, Govea-Salas M. Importance of Certain Varieties of Cucurbits in Enhancing Health: A Review. Foods. 2024;13(8):1142. 38672815 10.3390/foods13081142 PMC11048896

[58] Rolnik A, Olas B. Vegetables from the Cucurbitaceae family and their products: Positive effect on human health. Nutrition. 2020;78:110788. doi: 10.1016/j.nut.2020.110788 32540673

[59] Patel S, Rauf A. Edible seeds from Cucurbitaceae family as potential functional foods: Immense promises, few concerns. Biomed Pharmacother. 2017;91:330–7. doi: 10.1016/j.biopha.2017.04.090 28463796

