# Supplementary material for: Evaluation of Sub-acute toxicity and safety profile of Charmagaz seed oil in rats
Source: PLoS One. 2025 Jul 11;20(7):e0327697. doi: 10.1371/journal.pone.0327697 (PMC12250630; doi:10.1371/journal.pone.0327697)
Supplement: S1 Fig — (PDF) [file pone.0327697.s001.pdf]

INDUSTRIAL ANALYTICAL CENTRE

International Centre For Chemical And Biological Sciences

Data file: E:\DATA\FATTYACID_COMPOSITION_CPSIL-88\17869 2021-08-09 14-19-49\17869-52.D

**Instrument:**	7890B-GC	**Acq. operator:**	SYSTEM
**Sample name:**	17869-5	**Sample type:**	Unspecified
**Location:**	206	**Injection:**	2 of 2
**Injection vol.(uL):**	1.000	**Injection date:**	8/10/2021 4:37:35 AM
**Acq. method:**	AOCS-Ce-1h CP-Sil-88 100M Actual.M		

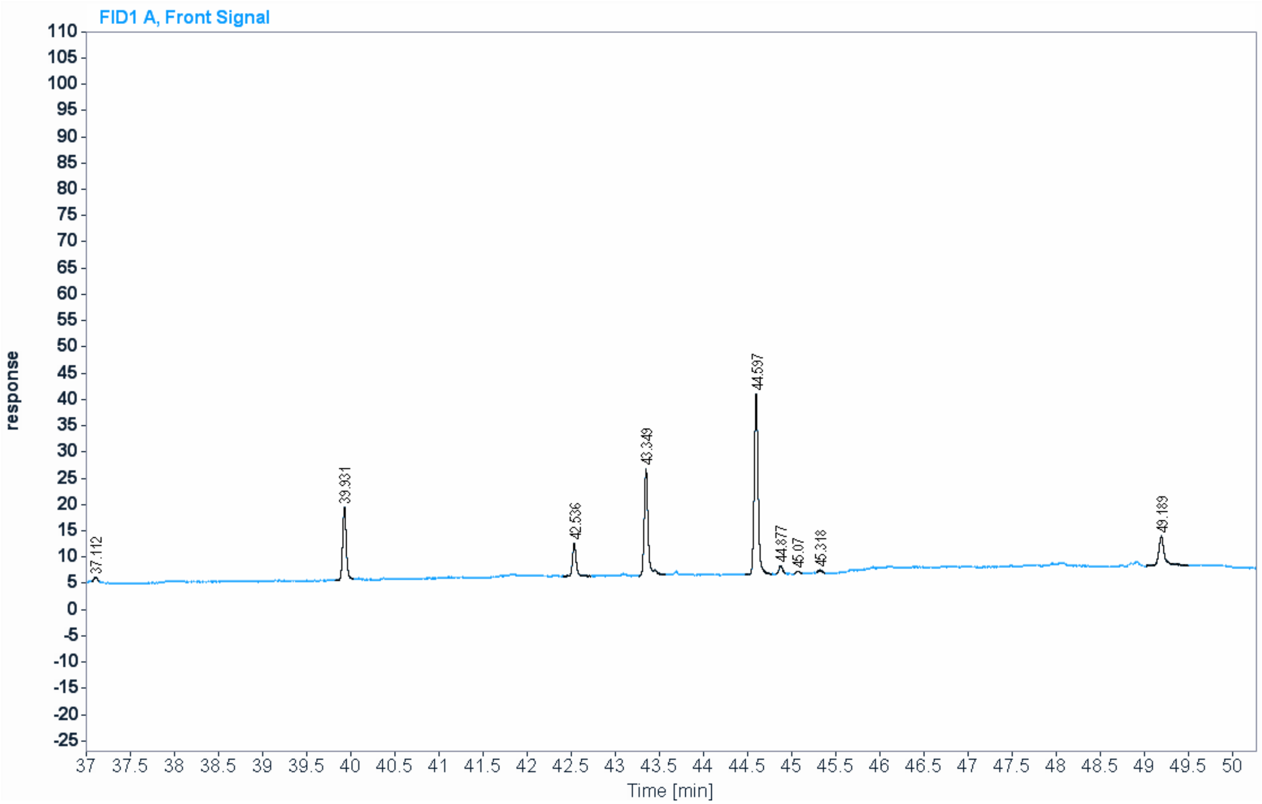

Signal: FID1 A, Front Signal

Peak #	Name	RT [min]	Height	Area	Area%
1	C14:0 Myristic acid	37.112	0.91	2.22	0.816
2	C16:0 Palmitic acid	39.931	13.94	39.58	14.514
3	C18:0 Stearic acid	42.536	6.34	20.19	7.402
4	C18:1 Oleic acid	43.349	20.48	65.41	23.988
5	C18:2 Linoleic acid(Trans)	44.597	34.48	107.15	39.293
6	C18:2 Linoleic acid(cis)	44.877	1.58	4.40	1.612
7	C20:0 Eicosanoic acid	45.070	0.55	1.65	0.606
8	C18:3 n-6 Linolenic acid	45.318	0.73	2.31	0.849
9	C22:1 Erucic acid	49.189	5.76	29.78	10.920
			Total	272.69	100.000

Analyzed by: ____________________ **Checked by:** ____________________
