# Supplementary material for: Evaluation of Sub-acute toxicity and safety profile of Charmagaz seed oil in rats
Source: PLoS One. 2025 Jul 11;20(7):e0327697. doi: 10.1371/journal.pone.0327697 (PMC12250630; doi:10.1371/journal.pone.0327697)
Supplement: S1 Table — (DOCX) [file pone.0327697.s002.docx]

| **Brine Shrimp Bioassay** | | | | |
| --- | --- | --- | --- | --- |
| **Name** | **Dose (µg/ml)** | **No. of Shrimps** | **No. of Survivors** | **% Mortality** |
| Etoposide | 7.5 | 30 | 9 | 70% |
| Charmagaz seed oil | 10 | 30 | 30 | 0% |
| Charmagaz seed oil | 100 | 30 | 26 | 13.34% |
| Charmagaz seed oil | 1000 | 30 | 26 | 13.34% |

**S1 Table: Percentage mortality of brine shrimps by Charmagaz seed oil**
